# Syringeless Electrospinning toward Versatile Fabrication of Nanofiber Web

**DOI:** 10.1038/srep41424

**Published:** 2017-01-25

**Authors:** Seongjun Moon, Manjae Gil, Kyung Jin Lee

**Affiliations:** 1 Department of Chemical Engineering and Applied Chemistry, College of Engineering, Chungnam National University, 99 Daehak-ro (st), Yuseong-gu, Daejeon, 305-764, Republic of Korea

## Abstract

Although electrospinning is considered a powerful and generic tool for the preparation of nanofiber webs, several issues still need to be overcome for real-world applications. Most of these issues stem from the use of a syringe-based system, where the key factor influencing successful electrospinning is the maintenance of several subtle balances such as those of between the mass and the electrical state. It is extremely difficult to maintain these balances throughout the spinning process until all the polymeric solution in the syringe has been consumed. To overcome these limitations, we have developed a syringeless electrospinning technique as an alternative and efficient means of preparing a nanofiber web. This new technique uses a helically probed rotating cylinder. This technique can not only cover conventional methods, but also provides several advantages over syringe-based and needless electrospinning in terms of productivity (6 times higher) and processibility. For example, we can produce nanofibers with highly crystalline polymers and nanofiber-webs comprising networks of several different polymers, which is sometimes difficult in conventional electrospinning. In addition, this method provides several benefits for colloidal electrospinning as well. This method should help expand the range of applications for electrospun nanofiber webs in the near future.

Electrospinning is a well-established technique for preparing nanofiber structures, and many studies have addressed the preparation and application of nanofiber webs^1^,^2^. Although the first patent related to electrospinning was filed in 1902^3^, detailed discussion of electrospinning did not begin to appear until the early 1990s as a result of advances in nanoscience^4^. The basic science of electrospinning and several techniques enabling the efficient preparation of nanofibers were reported in the early part of this century. Electrospun nanofibers have been applied to various fields that can leverage their characteristic properties (high surface area, adjustable pore volume, and broad range of material types)^5^,^6^,^7^, such as tissue engineering^8^,^9^,^10^,^11^,^12^,^13^,^14^,^15^, sensors^16^,^17^, drug delivery^18^,^19^,^20^, wound dressing^21^,^22^,^23^,^24^,^25^, and energy applications^26^,^27^,^28^.

Despite the attractive advantages of electrospinning, such as the simple procedure and material diversity, examples of the use of electrospinning in real-world applications have been limited, mainly because of the high cost of the product^29^,^30^. The electrospinning procedure is basically dependent on fine-tuning to maintain two balances: the balance between the elongation forces induced by electrostatic force and the surface tension of a polymer droplet and the competition between the rate of solidification by solvent evaporation and the rate of sphere formation by surface tension. In general electrospinning, the polymeric solution is delivered by a syringe pump, and the procedure can be considered a continuous one. Therefore, it is challenging to maintain these delicate balances during the entire procedure given that it is influenced by numerous parameters, including the properties of the polymeric solution (viscosity, conductivity, molecular weight of the polymer, concentration of the polymer, etc.), the experimental parameters (voltage, flow rate, distance between needle and collector), and the environmental parameters (humidity, temperature, etc.)^31^,^32^. These challenges are regarded as major hurdles to the mass production of nanofibers by electrospinning.

Herein, we report a modified syringeless electrospinning technique using a rotating, helically probed cylinder. This technique provides a facile and versatile means of preparing an electrospun nanofiber web. Conductive probes, the diameters of which could be between 0.1 mm and 1 mm, were integrated into a rotating cylinder and provided with a DC voltage supply to enable the easy drawing of a polymeric solution, thus generating a Taylor cone. These modifications make the electrospinning process a batch-based continuous system, and thus avoid suffering from tedious optimization procedure. With this syringeless system, results identical to those of conventional electrospinning can be realized relatively easily. In addition, several technical breakthroughs are proposed to overcome difficulties presented by the conventional electrospinning procedure.

## Results and Discussion

### Difficulties with Conventional Electrospinning Procedures

The first reason for the limited take-up of the electrospinning process by industry is the high cost of electrospun nanofiber webs. Although it is relatively simple to produce nanofibers on a laboratory scale, it is very challenging to scale this up to large-scale continuous processing. It is also true that the electrospinning process is more generic than other nanofiber fabrication methods. However, in actual applications, electrospinning incurs relatively higher production costs than the process for producing spun nanofibers because the process is dependent on various parameters that directly or indirectly affect the production cost, such as the concentration of the polymeric solution, applied voltage, and solution feed rate. In addition, the control of one factor can cause changes to other factors, and thus optimizing the process conditions is relatively difficult. Therefore, the electrospinning process lacks price competitiveness as compared to other spinning techniques for the fabrication of low-functional nanofibers.

Secondly, in terms of material diversity, electrospinning should support the use of a wide range of polymers such as poly(methyl methacrylate) (PMMA), polyacrylonitrile (PAN), poly(vinyl alcohol) (PVA), and poly(lactic-co-glycolic acid) (PLGA). However, in reality, the selection of the polymer is subject to many limitations. For example, it is well known that polymers exhibiting a high degree of crystallinity are difficult to adopt for the electrospinning procedure because solvent evaporation tends to lead to nozzle blocking. There have been several outstanding reports describing means of overcoming this drawback, most of which are based on core/shell electrospinning techniques^33^,^34^. In addition, the entanglement of the polymeric chains in a solution is crucial to the electrospinning process because the polymer droplets should explode into smaller droplets in the vicinity of Rayleigh’s limitation as a result of fiber thinning by the application of an electrical potential. This means that the use of a polymeric solution with a relatively high concentration is inevitable for the spinning of nano-fibers. Therefore, polymers with low solubility such as those with electrical properties or those which form globular structures in solution (such as proteins or DNA) are difficult to apply to electrospinning. Polymers with a low molecular weight or functional oligomers are also difficult to adopt for the same reason.

Mass production and material selection are the two main issues that need to be addressed to enable the versatile application of electrospun nanofiber webs. We think that these two issues are attributable to the use of a syringe-based system. In electrospinning, the Taylor cone should be stably maintained during the entire procedure. Although several successful results have been reported to date, it is clear that a stable Taylor cone is difficult to obtain because many hydrodynamic forces are acting during the electrospinning process. For example, it is necessary to control the mass balances in the Taylor cone, which is dependent on the feed rate and production rate of the fibers. The production rate of the fibers is itself affected by the applied electrical potential, conductivity, and viscosity of the polymeric solution. The viscosity of the solution is also influenced by the concentration, the theta (θ) condition of the polymeric solution, the molecular weight of the polymers, etc. The surface tension and solidification rate of the electrospun jet are also important parameters. The solidification rate is also influenced by environmental conditions such as the temperature and humidity. The Taylor cone must be finely controlled by maintaining the values of numerous experimental parameters during the entire procedure. This is extremely difficult to achieve in practice, and it is this which has prevented the widespread take-up of the electrospinning process. Practically, therefore, the adoption of a syringe-based continuous system is impossible.

Recent research into needleless electrospinning has provided an alternative pathway and improved the possibility of the mass production of electrospun nanofiber webs. Some researchers have reported on needleless electrospinning systems using rotating disks^35^, rollers^36^, balls^37^, and bubbles^38^ to obtain huge amounts of nanofibers. The most striking advantage of needleless electrospinning is that the spinning process is now dependent on the formation of numerous small droplets on the surface of the drum or disk/coil. Therefore, it is not necessary to maintain Taylor cones throughout the entire process, and thus the procedure can be used to realize a batch process. One limitation of this approach is that the surface energy difference between the surfaces of the rotation drum (or coils) and polymeric solution must be considered in each case. Of crucial importance for needless electrospinning is the generation of polymeric droplets on the flat surface of the rotating drum (or coils). For example, when one tries to make nanofibers with a water-based polymeric solution, a hydrophobic surface should be provided, which limits the choices available for the type of polymeric solution. Secondly, very high voltages are required to generate a Taylor cone of polymeric droplets on a flat surface. Considering the basic mechanism of nanofiber generation using electrospinning, a DC voltage applied to a polymeric droplet, causes the charge to migrate to the surface of the droplet. When an additional charge is applied, the droplet will try to increase its surface area to contain more charges on its surface. This means that when the polymeric solution is applied to a flat surface, some energy from the DC voltage will be consumed in an effort to generate spherical droplets. When more charges are applied, the droplet will try to explode into smaller droplets in order to increase its surface area through Taylor cone generation. When there is enough entanglement between polymer chains in the solution, however, the droplet cannot explode, and nanofibers form from the end of the cone. However, if one can start with a spherical droplet (or small droplets), less charge will be required to generate a Taylor cone. Therefore, the syringeless method, which incorporates needles on a rotating drum, can be operated at lower applied voltages than needless electrospinning (such as coil electrospinning and rotating drum electrospinning). In addition, the need for high voltages is also undesirable from the viewpoint of energy consumption. Even more importantly, the use of a high voltage increases the chance of spark generation in a system. Therefore, any solvent with a low flash point would be difficult to adopt for use in needless electrospinning, meaning that most of the powerful solvents such as chloroform, tetrahydrofuran (THF), and toluene cannot be used as a base solvent.

**Figure 1:**
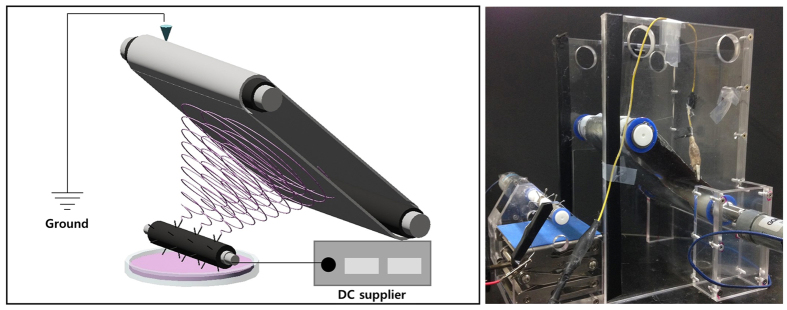
Schematic diagram and realistic electrospinning machine of rotating probed cylinder.

We attempted to integrate several needles into the rotating drum to reduce the applied voltage, as shown in Fig. 1. A rotating cylinder with several needles integrated into the surface was connected to a DC power supply, and the polymeric solution was positioned under the rotating cylinder. A positive voltage was applied to the needles, and the cylinder was rotated at the desired speed. Polymer droplets formed at the end of each needle, and a Taylor cones was generated on each needle when a positive voltage was applied. The resulting nanofibers were drawn to the grounded collector until the small droplets were consumed.

### Electrostatic effect between needles on helically probed rotating cylinder and on general probed cylinder

With the introduction of needles into the system, the issues facing mass production can be revisited. In conventional, syringe-based electrospinning, the mass production of nanofibers is challenging because any increase in the number of needles will have an adverse influence on the system because of the interaction between the static electricity on the needles^39^,^40^,^41^. In general, a nonwoven nanofiber mat is formed by a whipping motion of the electrospun nanofibers after jetting of the polymeric solution. However, when the needles are too close together, the electrostatic interaction will affect the free whipping motion. Therefore, it is difficult to obtain fine nanofibers or nanofiber mats using conventional, syringe-based electrospinning.

**Figure 2:**
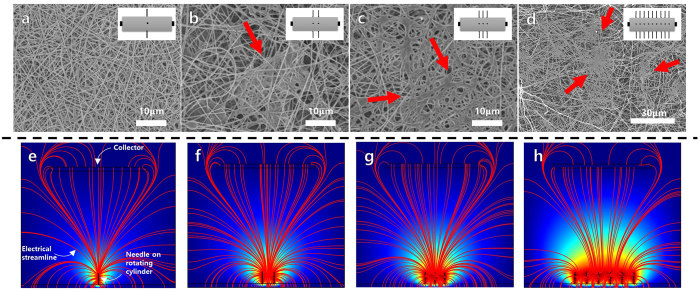
SEM images of PAN nanofibers fabricated by syringeless electrospinning with different number of needle on rotating drums. Insets were the type of the rotating cylinders with (**a**) single needle, (**b**) dual needles, (**c**) triple needles, and (**d**) multi-needles. (**e**~**h**) represents electrical streamline simulation results according to number of needle.

Figure 2a–d show SEM images of electrospun PAN nanofiber mats fabricated by syringeless electrospinning with different numbers of needles on the rotating cylinder (inset: diagram of rotating cylinders and needle configuration). The polymeric solution was 9% PAN in dimethylformamide (DMF). Although PAN nanofibers were successfully obtained in all cases, the morphology of the nanofiber mats was destroyed as the number of needles on the rotating cylinder increased. In addition, the number of aggregates observed on the nanofiber mats increased as the number of needles increased. In Fig. 2d (multi-needle configuration), the nanofiber morphology can barely be observed.

This phenomenon indicates that the electrostatic interaction between the needles, which increases as the number of needles on the cylinder increases (that is, as the distance between the needles decreases), adversely affects the morphology of the nanofiber web. To confirm these phenomena theoretically, the electrostatic streamlines that occur during electrospinning were calculated using Comsol. As shown in Fig. 2e–h, the electrical streamlines for the dual-, triple-, and multi-needle configurations were clearly not uniform, and some of the electrical streamlines failed to reach the collector. This can be attributed to the destruction of the morphologies of the nanofibers because the electrical force could not focus on the collector.

Furthermore, a relatively large number of overlapped fields appeared as the number of needles increased, resulting in the non-effective transport of the electrical potential onto the fiber jet. In general, after a polymeric solution was jetted from the needle, the resulting nanofiber traveled to the collector with a whipping motion. However, when overlapping of the electrical field occurred, its influence interfered with the free-jetting motion of the polymeric fiber. Repulsive forces were induced between the nanofibers, resulting in the destruction of the fiber morphology. Therefore, the distance between the needles must be large enough to produce a clear nanofiber morphology. However, any increase in the needle distance led to a decrease in the production rate because there were fewer needles on the cylinder.

**Figure 3:**
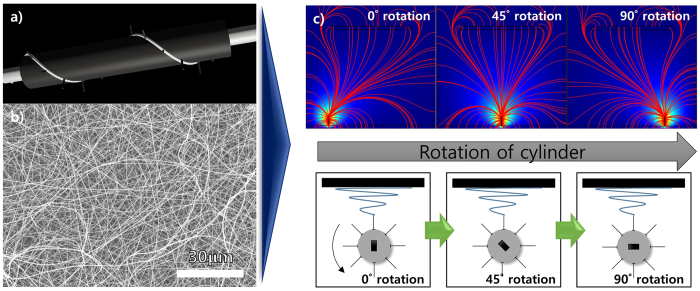
(**a**) Schematic diagram of helical probed cylinder, (**b**) SEM data of PAN nano-fiber using helical probed rotating cylinder and (**c**) computer simulating data according to rotation of the cylinder.

To overcome these issues, i.e., that a large number of needles is required for productivity but the same configuration causes destruction of the fiber morphology, needles were added to a rotating cylinder in a helical configuration. Figure 3a–c show a schematic of a helically probed cylinder, the morphology of a PAN nanofiber mat produced by this system, and the electrical streamlines forecast by Comsol (bottom: schematic of rotating cylinder during process), respectively. The needles on the cylinder were configured helically and a conductive silver paste (white) was used to connect the needles. As compared to the SEM images shown in Fig. 2, the morphology of the nanofiber mat produced by the helically probed cylinder was relatively uniform, and the productivity was comparable to that of the multi-needle cylinders. This result means that mass production without morphological destruction of the nanofibers can be achieved by syringeless electrospinning with a helically probed rotating cylinder. With this configuration, although the needles are close enough to induce an electrical interaction between them, the majority of the interaction arises from the needles closest to the collector. Therefore, the electrostatic effect occurs at the top of needles merely by rotating the cylinder. As shown in Fig. 3, not all needles interacted with the collector simultaneously. Rather, only one needle generated an electrical streamline toward the collector during cylinder rotation. These results are representative of the helically probed rotating cylinder, and thus it is possible to prepare polymeric nanofibers with a higher degree of uniformity and good distribution. In addition, when polymeric droplets are generated on top of the needles, it is possible to cause complete consumption of the droplets by controlling either the rotational speed of the cylinder or the electrical potential.

### Versatile selection of polymeric solution

**Figure 4:**
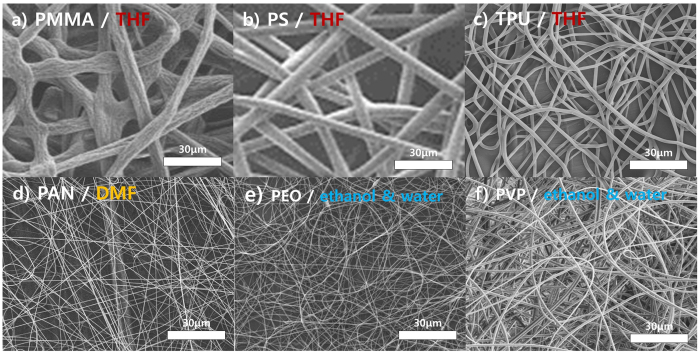
SEM characterization of nanofiber fabricated by electrospinning process of rotating probed cylinder using variety of polymers and solvents ((**a**) PMMA/THF, (**b**). PS/THF (**c**) TPU/THF, (**d**) PAN/DMF, (**e**) PEO/ethanol and water, (**f**) PVP/ethanol and water).

In the case of needleless electrospinning, the overriding factor is the type of polymeric solution because the polymeric droplets must easily form on the rotating cylinders^42^,^43^,^44^. Therefore, selection of the polymeric solution depends strongly on the surface characteristics of the cylinders, including the surface pattern of the cylinder, the attraction between the polymeric solution and the cylinder surface, and the concentration of the solution. Therefore, the selection of polymers and solvents for needleless electrospinning is very restrictive. However, there are fewer limitations with the syringeless method using helically probed needles for the fabrication of a nanofiber web. Figure 4 shows SEM images of diverse polymer nanofibers produced by syringeless electrospinning with various combinations of polymers and solvents. Regardless of the solvent used, including organic and aqueous solvents, polymeric nanofibers were fabricated with high uniformity and good morphology. Examples include PMMA dissolved in THF (Fig. 4a), PAN in DMF (Fig. 4d), poly(ethylene oxide) (PEO) in ethanol (Fig. 4e), polystyrene (PS) in THF (Fig. 4b), thermoplastic polyurethane (TPU) in THF (Fig. 4c), and polyvinyl pyrrolidone (PVP) in water (Fig. 4f). These results demonstrate that polymeric nanofibers are more easily obtained with the helically probed rotating system than with a general needleless electrospinning process. Although all the nanofiber webs shown in Fig. 4 were obtained under similar experimental conditions except for the type of polymeric solution, nanofiber webs were successfully prepared in all cases, meaning that optimization with the syringeless system is much easier than with a syringe-based system. This diversity is comparable to that of general electrospinning with a syringe-based system except that with the syringeless method, it is easier to optimize processing conditions because each droplet plays a role in numerous batch-reactors, as described above^31^,^32^. Furthermore, although the needleless electrospinning method requires a high supply voltage of 20 to 60 kV to fabricate the nanofibers^42^,^43^,^44^, the current modified method requires only 15 to 17 kV. Because the voltage required to generate the Taylor cone is lower than that in needleless electrospinning, the process is relatively safe, allowing a wider range of solvent selection.

In Fig. 4, although nanofiber webs with various combinations of polymers and solvents were successfully prepared, there were some variations in the fiber thickness. However, the thickness of the nanofibers can be controlled in syringeless electrospinning using a method similar to that adopted for syringe-based electrospinning. In electrospinning science, the thickness of the resulting nanofibers is governed by a broad range of experimental parameters, such as the concentration of the polymeric solution, the applied voltage, and the type of solvent. Supplementary Figure S1 shows an SEM image of PVP nanofibers produced with a range of concentrations of a polymeric solution in ethanol with water as the co-solvent. As depicted in the image, the diameter of the nanofibers was controlled to between 2 μm and 800 μm by adjusting the concentration.

**Figure 5:**
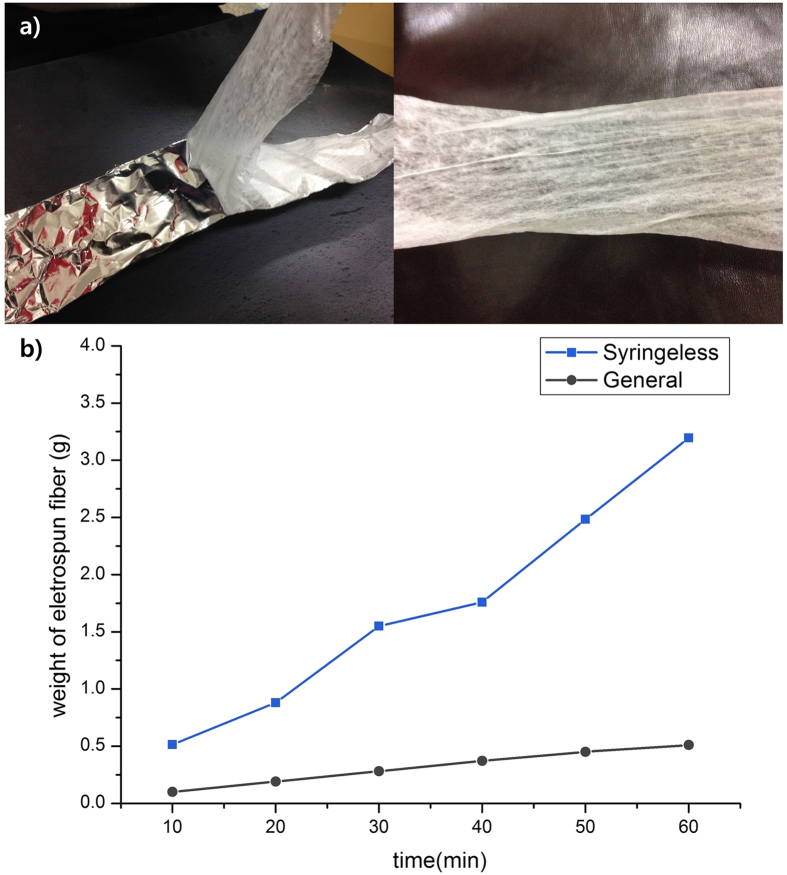
(**a**) One step mat-formed electrospun nonwoven prepared via helically probed cylinder system (TPU nano-fiber as polymer in THF as solvent). (**b**) Plot of weight of electrospun fibers of general electrospinning method and our syringeless system.

Syringeless electrospinning with a helically probed cylinder not only provides material versatility and controllable results but also realizes a higher level of productivity than is possible with the syringe-based system. Figure 5a is a photograph of a TPU nanofiber mat produced by lab-scale helically probed syringeless electrospinning (Fig. 1, right). A nanofiber mat was successfully prepared and the productivity rate was approximately 3.2 g/h, which is six times higher than that of syringe-based electrospinning with a single nozzle, as shown in Fig. 5b. Therefore, the helically probed rotating cylinder can be used for the mass production of a nanofiber web using a wide range of polymeric solutions.

### Fabrication of PVDF nanofiber

**Figure 6:**
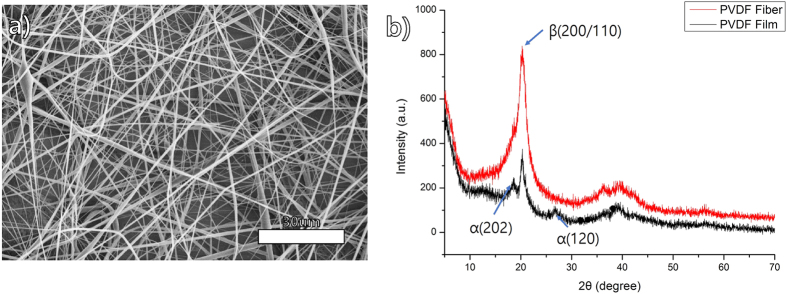
(**a**) SEM image of electrospun PVDF nano-fiber prepared using helically probed rotating cylinder, (**b**) XRD data of the PVDF nano-fiber (red) and pristine film (black).

Syringeless electrospinning has several advantages over needleless or syringe-based systems, including high productivity and material and solvent diversity. In addition, this system is basically a batch-based continuous system, unlike the syringe-based system, and thus it provides easy accessibility to the process. Several polymers that cannot be used for electrospinning can now be handled relatively easily by syringeless electrospinning. The semi-crystalline polymer polyvinylidene fluoride (PVDF) has attracted the attention of many researchers because of its nonlinear optical and electroactive properties, strong corrosive resistance, good electric susceptibility, and high dielectric constant^45^,^46^,^47^. Because of its characteristic properties, PVDF has been adopted for many applications such as sensors, electrical devices, and membranes. The polymer consists of four crystalline phases, namely, the α, β, γ, and δ phases. Generally, the α phase is the most abundant phase, and it can be obtained commercially in both powder and pellet form, whereas the β, γ, and δ phases can only be obtained by further processing of α-phase PVDF. Among these, the β phase of PVDF has attracted particular attention in the area of electrospinning because PVDF nanofibers have characteristic electrochemical properties such as piezoelectricity^48^. However, in terms of the production of PVDF nanofibers via electrospinning, it has been a challenge to obtain continuous nanofibers, mainly because of their crystallinity but also because of their solubility. The orifice of the needle often gets blocked by the polymeric solution over time because of crystal formation. Therefore, the general electrospinning processes are not suitable for the mass production of PVDF nanofibers. However, PVDF β-phase nanofibers were readily prepared by helically probed syringeless electrospinning. Figure 6a shows the morphology of a PVDF nanofiber produced by the helically rotating cylinder process. The crystal phases of an electrospun PVDF nanofiber and a pristine PVDF nanofilm were confirmed via XRD spectroscopy (Fig. 6b). Fine nanofiber mats with fibers of a uniform diameter were obtained successfully. In addition, the XRD data shows that the electrospun PVDF nanofiber contained β-phase crystallinity, as described in previous reports addressing syringe-based electrospinning. It is well known that the β phase is abundant in electrospun PVDF nanofibers because of the stretching caused by the electrostatic force in the electrospinning process, which can change the phase separation^49^. Therefore, the new electrospinning method can be used to fabricate functional nanofibers relatively easily as compared to previous methods.

### Applications of helically probed rotating cylinder

**Figure 7:**
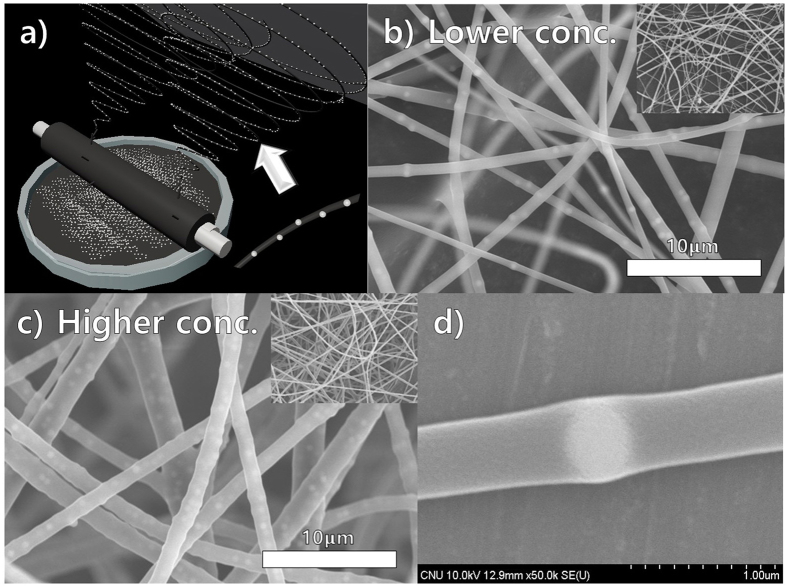
(**a**) Schematic diagram of colloidal electrospinning using helically probed rotating cylinder, (**b**) SEM images represented the colloidal electrospinning using PVP in water and ethyl alcohol with the lower concentration of 500 nm sized silica nano-particles (0.5 g/40 ml), (**c**) SEM images shows the colloidal electrospinning with high concertation (1.5 g/40 ml), (**d**) SEM image of single fibers having silica particles with high magnification.

Syringeless electrospinning with a helically probed rotating cylinder has the potential to solve several issues encountered in syringe-based systems. First, this method can to be applied to colloidal electrospinning, which is used to prepare polymeric nanofibers containing foreign colloidal particles to enhance their functionality^50^. The realization of a continuous colloidal electrospinning process with a massive number of particles in a syringe-based system is difficult because the high concentration of particles in the polymeric solution leads to the blocking of the needle and prevents the accurate generation of a Taylor cone. Therefore, there are certain limitations in terms of the number and size of colloidal particles that can be used in colloidal electrospinning^51^. However, the number and size of colloidal particles are not crucial parameters in helically probed syringeless electrospinning. Figure 7a show a schematic of colloidal electrospinning using the syringeless technique; Fig. 7b and c show SEM images of PVP nanofibers containing 500-nm silica particles in low and high concentrations, respectively. A nanofiber mat was successfully obtained using a helically probed rotating cylinder in which continuous processing was possible. Furthermore, fibers with a highly uniform diameter and a homogeneous dispersion of nanoparticles were obtained. By increasing the concentration of the particles, the diameter of the fibers was increased, as was the number of particles in those fibers (Fig. 7b). In addition, as indicated in Fig. 7d, colloidal particles were successfully incorporated into the polymeric nanofibers. Although a sedimentation issue can occur as part of the process, this issue can be overcome by external stirring of the solution.

**Figure 8:**
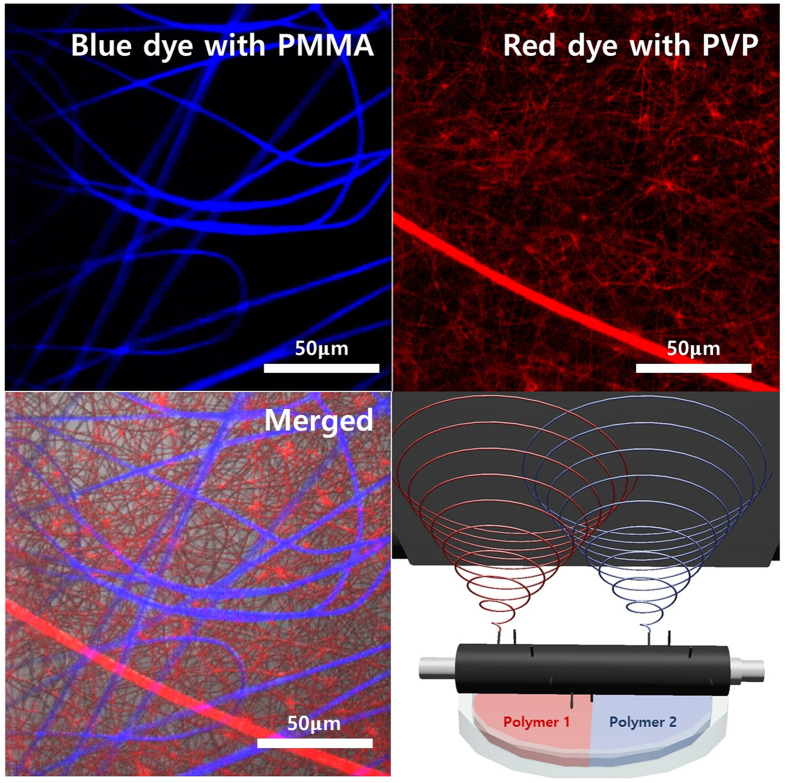
CLSM image shows the multilayered fiber mat using and PMMA in THF with Poly [(m-phenylenevinylene)-co-(2, 5-dioctoxy-p-phenylenevinylene)] as blue dye and PVP in ethanol with rhodamine as red dye and schematic diagram about multi-layered mass electrospinning,.

Second, syringeless electrospinning provided a powerful platform for the production of a multi-layered electrospun mat with different combinations of materials (Fig. 8). In general, with most electro-spinning processes, including syringe-based electrospinning and needleless electrospinning, it is very challenging to produce nanofiber webs composed of different materials on the same collector at the same time because of the limitations of the apparatus and the electrostatic interaction between the nanofibers. The helically probed cylinder can overcome these limitations. As confirmed by the confocal laser scanning microscopy (CLSM) data, the helically probed cylinder can generate a nanofiber web network with different materials simply by dividing the feed container as shown in Fig. 8. The solution container can be divided into two parts, with each part being filled with different polymeric solutions (here, PVP in ethanol and PMMA in THF). These results demonstrate that the helically probed needles on the cylinder prevent interaction between the nanofibers; the needles act individually because the probe needles are located so far from each other when the polymeric solution is jetted from the needle. This method can be extended to prepare more functional nanofiber webs for other applications.

## Conclusions

Syringeless electrospinning, an advanced and highly versatile technique, was successfully demonstrated using a rotating helically integrated cylinder. This method was proven to be superior to general electrospinning and needleless electrospinning in terms of processibility, selection of materials and solvent, and productivity. The helically integrated needles in the cylinder provide not only mild electrospinning conditions but also several advantages for overcoming many of the limitations facing mass production with electrospinning. Furthermore, this method can be extended to prepare more functional nanofiber webs and, when combined with other techniques such as colloidal electrospinning, the preparation of nanofiber webs with several different materials. For example, the preparation of nanofibers with a conducting polymer can be realized. This syringeless electrospinning procedure will contribute to the mass production of nanofiber webs and could lead to finding new applications for nanofibers in biological, chemical, and medical fields.

## Methods

### Apparatus for syringe-free electrospinning

To fabricate the helically probed rotating cylinder, Teflon cylinder which has 10 mm of diameter and 150 mm of length was selected as a framework. 12 Conductive needles which has 10 mm of length were helically impregnated at intervals of 5 mm on center of the framework. On the side, the needles were located on the cylinder every 45°. Every needles were linked using silver paste and the end of paste was connected with high voltage supplier to supply the electrical potential. To supply the polymeric solution to the needles, the glass dish was located under the rotating cylinder. And then, depending on the rotation of the cylinder, the polymeric solutions were plunged onto the needles. In addition, to obtain the fibers which were generated by the apparatus, the conductive collector was located upper the rotating cylinder and connected with a ground. The rotating rpm of the collect was 30 rpm. The tip to collector distance was fixed from 150 mm to 200 mm.

### Polymer solution preparations

PAN polymer (Mw = 150 000 g/mol, Aldrich) was dissolved in DMF (99.8%, Aldrich) to produce 8, 9, and 10 wt% solutions. PMMA (Mw = 120 000 g/mol, Aldrich), PS (Mw = 280 000 g/mol), and TPU (Neothane, DongSung corp.) were dissolved in THF as solvent to produce 6, 8, and 10 wt% polymeric solutions, respectively. And thus, PEO (Mv = 200 000, powder, Aldrich) and PVP (Mw = 360 000, Aldrich) were dissolved in a solution including 75 v% of ethyl alcohol (Aldrich) and 25 v% of distilled water for production of 10, 12.5, 15, 17.5, 22.5, and 25 wt% solutions respectively. To obtain PVDF nano fiber, PVDF (Mw ~275 000 by GPC, Aldrich) was dissolved as 24 wt% in 50% of acetone and 50% of DMF mixture.

### Preparation of nano-fibers using polymeric solution

The nano-fibers were obtained using the probed rotating cylinder machines. The machine includes a solution supplier, the probed cylinder connected with rotating motor, rolling collector, and DC voltage supplier. The polymer solutions which located on the solution supplier was loaded onto the surface of the needles by the slow rotation of the cylinder. The rotating speed was controlled as 50 rpm. The polymeric solution travel to the collector which was covered with aluminum foil by supplied electricity. The supplied voltage and distance between the collector and the cylinder was set as 15~19 kV, and 150 mm respectively. And then, all prepared nano-fibers were determined by SEM (S-4800 from Hitachi).

### Colloidal electrospinning

The solution for colloidal nano-fiber was prepared using PVP polymeric solution and silica nano particle. The PVP polymer was dissolved in the mixture including 25 v% of distilled water and 75 v% of ethyl alcohol to produce 15 wt% polymer solution. The silica nano particle as colloidal particle was prepared by Stöber method and its controlled diameter was 500 nm. The solution and the particle were mixed using ultra-sonicator and magnetic bar to prepare 1.25 and 2.5 wt% colloidal solutions. The mixed solutions were loaded onto the surface of needles on the cylinder and electro-spun using the rotating machine.

### Multilayered electrospinning

the multi-layered polymer mat was fabricated using different polymer solutions including different fluorescence dyes. Rhodamine B isothiocyanate, and Poly [(m-phenylenevinylene)-co-(2, 5-dioctoxy-p-phenylenevinylene)] were used as the dyes to represent red, and blue colors respectively. The multi-layered electrospinning was proceeded using solution supplier with divided section. The solutions were poured on the divided section respectively, and loaded onto the helically probed cylinder. The connection of the needles on the cylinder with DC voltage supplier, and the rotation of the cylinder induced the fabrication of the multi-layered nano-fiber mat. And then, the mats were confirmed by Confocal Laser Scan Microscope (LSM5 LIVE from Carl Zeiss).

## Additional Information

**How to cite this article**: Moon, S. *et al*. Syringeless Electrospinning toward Versatile Fabrication of Nanofiber Web. *Sci. Rep.* **7**, 41424; doi: 10.1038/srep41424 (2017).

**Publisher's note:** Springer Nature remains neutral with regard to jurisdictional claims in published maps and institutional affiliations.

## Supplementary Material

Supporting Information
