# Supplementary material for: Syringeless Electrospinning toward Versatile Fabrication of Nanofiber Web
Source: Sci Rep. 2017 Jan 25;7:41424. doi: 10.1038/srep41424 (PMC5264178; doi:10.1038/srep41424)
Supplement: Supporting Information [file srep41424-s1.doc]

**Supplementary information**

# Syringeless Electrospinning toward Versatile Fabrication of Nanofiber Web

Seongjun Moon^1.+^ , Manjae Gil^1,+^, and Kyung Jin Lee^1^,*

^1^Department of Chemical Engineering and Applied Chemistry, College of Engineering, Chungnam National University, 99 Daehak-ro (st), Yuseong-gu, Daejeon, 305-764, Republic of Korea

[*kjlee@cnu.ac.kr](mailto:*kjlee@cnu.ac.kr)


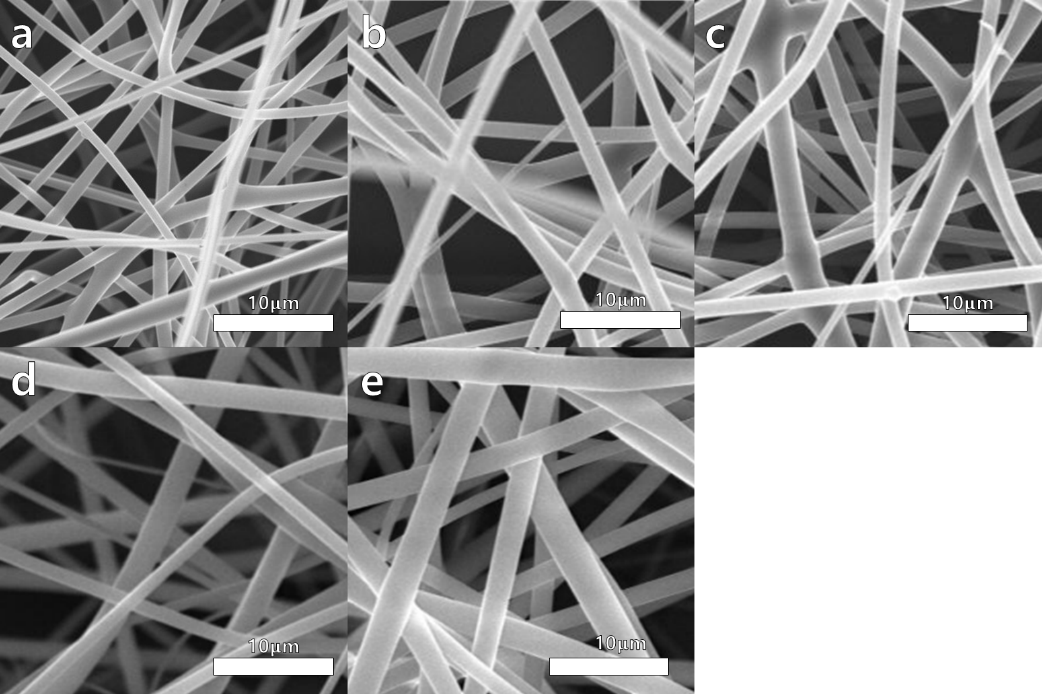


**Supplementary Figure S1.** SEM images about controlling diameter of PVP nanofibers according to concentration of polymer solutions (a. 5 g, b. 6 g, c. 7 g, d. 9 g, e. 10 g per 30 ml of ethanol with 10 ml of water.
